# Supplementary material for: Pyruvate Dehydrogenase Kinase 1 inhibition mediated oxidative phosphorylation enhancement in cartilage promotes osteoarthritis progression
Source: BMC Musculoskelet Disord. 2023 Jul 20;24:597. doi: 10.1186/s12891-023-06585-6 (PMC10357736; doi:10.1186/s12891-023-06585-6)
Supplement: Supplementary file 1 — Additional file 1. [file 12891_2023_6585_MOESM1_ESM.zip › BP.Gsea.1653623667859/GOBP_PLACENTA_DEVELOPMENT.html]

Details for gene set GOBP\_PLACENTA\_DEVELOPMENT[GSEA]

|  || Dataset | 55457\_collapsed\_to\_symbols.cls.cls #OA\_versus\_normal.cls.cls #OA\_versus\_normal\_repos |
| Phenotype | cls.cls#OA\_versus\_normal\_repos |
| Upregulated in class | normal |
| GeneSet | GOBP\_PLACENTA\_DEVELOPMENT |
| Enrichment Score (ES) | -0.3823845 |
| Normalized Enrichment Score (NES) | -1.7082653 |
| Nominal p-value | 0.0 |
| FDR q-value | 0.12875295 |
| FWER p-Value | 1.0 |
Table: GSEA Results Summary

  

Fig 1: Enrichment plot: GOBP\_PLACENTA\_DEVELOPMENT      
 Profile of the Running ES Score & Positions of GeneSet Members on the Rank Ordered List

  

| SYMBOL | TITLE | RANK IN GENE LIST | RANK METRIC SCORE | RUNNING ES | CORE ENRICHMENT || 1 | RTCB | "RNA 2',3'-cyclic phosphate and 5'-OH ligase [Source:HGNC Symbol;Acc:HGNC:26935]" | 78 | 0.947 | 0.0163 | No |
| 2 | PTN | pleiotrophin [Source:HGNC Symbol;Acc:HGNC:9630] | 402 | 0.706 | 0.0077 | No |
| 3 | SPINT2 | "serine peptidase inhibitor, Kunitz type 2 [Source:HGNC Symbol;Acc:HGNC:11247]" | 541 | 0.645 | 0.0121 | No |
| 4 | GJB3 | gap junction protein beta 3 [Source:HGNC Symbol;Acc:HGNC:4285] | 549 | 0.641 | 0.0267 | No |
| 5 | LDOC1 | LDOC1 regulator of NFKB signaling [Source:HGNC Symbol;Acc:HGNC:6548] | 989 | 0.520 | 0.0046 | No |
| 6 | AKT1 | AKT serine/threonine kinase 1 [Source:HGNC Symbol;Acc:HGNC:391] | 1192 | 0.478 | 0.0001 | No |
| 7 | CEBPA | CCAAT enhancer binding protein alpha [Source:HGNC Symbol;Acc:HGNC:1833] | 1750 | 0.392 | -0.0343 | No |
| 8 | ANG | angiogenin [Source:HGNC Symbol;Acc:HGNC:483] | 2009 | 0.360 | -0.0460 | No |
| 9 | TPPP3 | tubulin polymerization promoting protein family member 3 [Source:HGNC Symbol;Acc:HGNC:24162] | 2041 | 0.357 | -0.0400 | No |
| 10 | GRHL2 | grainyhead like transcription factor 2 [Source:HGNC Symbol;Acc:HGNC:2799] | 2111 | 0.348 | -0.0371 | No |
| 11 | PRDX3 | peroxiredoxin 3 [Source:HGNC Symbol;Acc:HGNC:9354] | 2161 | 0.343 | -0.0329 | No |
| 12 | KRT19 | keratin 19 [Source:HGNC Symbol;Acc:HGNC:6436] | 2258 | 0.331 | -0.0326 | No |
| 13 | MAPK14 | mitogen-activated protein kinase 14 [Source:HGNC Symbol;Acc:HGNC:6876] | 2352 | 0.320 | -0.0323 | No |
| 14 | SOD1 | superoxide dismutase 1 [Source:HGNC Symbol;Acc:HGNC:11179] | 2413 | 0.313 | -0.0296 | No |
| 15 | WNT2 | Wnt family member 2 [Source:HGNC Symbol;Acc:HGNC:12780] | 2700 | 0.279 | -0.0454 | No |
| 16 | SOX15 | SRY-box transcription factor 15 [Source:HGNC Symbol;Acc:HGNC:11196] | 2748 | 0.275 | -0.0426 | No |
| 17 | CDKN1B | cyclin dependent kinase inhibitor 1B [Source:HGNC Symbol;Acc:HGNC:1785] | 2984 | 0.251 | -0.0550 | No |
| 18 | SENP2 | SUMO specific peptidase 2 [Source:HGNC Symbol;Acc:HGNC:23116] | 3040 | 0.246 | -0.0535 | No |
| 19 | PCDH12 | protocadherin 12 [Source:HGNC Symbol;Acc:HGNC:8657] | 3081 | 0.242 | -0.0509 | No |
| 20 | TFEB | transcription factor EB [Source:HGNC Symbol;Acc:HGNC:11753] | 3190 | 0.232 | -0.0539 | No |
| 21 | TRIM28 | tripartite motif containing 28 [Source:HGNC Symbol;Acc:HGNC:16384] | 3526 | 0.200 | -0.0754 | No |
| 22 | VDR | vitamin D receptor [Source:HGNC Symbol;Acc:HGNC:12679] | 3571 | 0.197 | -0.0742 | No |
| 23 | CSF2 | colony stimulating factor 2 [Source:HGNC Symbol;Acc:HGNC:2434] | 3699 | 0.185 | -0.0798 | No |
| 24 | ST14 | ST14 transmembrane serine protease matriptase [Source:HGNC Symbol;Acc:HGNC:11344] | 3900 | 0.165 | -0.0916 | No |
| 25 | SPINT1 | "serine peptidase inhibitor, Kunitz type 1 [Source:HGNC Symbol;Acc:HGNC:11246]" | 3938 | 0.161 | -0.0906 | No |
| 26 | HEY2 | hes related family bHLH transcription factor with YRPW motif 2 [Source:HGNC Symbol;Acc:HGNC:4881] | 4058 | 0.150 | -0.0964 | No |
| 27 | SPP1 | secreted phosphoprotein 1 [Source:HGNC Symbol;Acc:HGNC:11255] | 4205 | 0.137 | -0.1046 | No |
| 28 | CDKN1C | cyclin dependent kinase inhibitor 1C [Source:HGNC Symbol;Acc:HGNC:1786] | 4233 | 0.134 | -0.1036 | No |
| 29 | VASH2 | vasohibin 2 [Source:HGNC Symbol;Acc:HGNC:25723] | 4345 | 0.124 | -0.1093 | No |
| 30 | IGF2 | insulin like growth factor 2 [Source:HGNC Symbol;Acc:HGNC:5466] | 4378 | 0.123 | -0.1089 | No |
| 31 | EPOR | erythropoietin receptor [Source:HGNC Symbol;Acc:HGNC:3416] | 4596 | 0.105 | -0.1234 | No |
| 32 | ALKBH1 | "alkB homolog 1, histone H2A dioxygenase [Source:HGNC Symbol;Acc:HGNC:17911]" | 4765 | 0.090 | -0.1345 | No |
| 33 | OVOL2 | ovo like zinc finger 2 [Source:HGNC Symbol;Acc:HGNC:15804] | 5017 | 0.071 | -0.1524 | No |
| 34 | BIRC2 | baculoviral IAP repeat containing 2 [Source:HGNC Symbol;Acc:HGNC:590] | 5054 | 0.069 | -0.1536 | No |
| 35 | ASCL2 | achaete-scute family bHLH transcription factor 2 [Source:HGNC Symbol;Acc:HGNC:739] | 5194 | 0.057 | -0.1632 | No |
| 36 | FZD5 | frizzled class receptor 5 [Source:HGNC Symbol;Acc:HGNC:4043] | 5392 | 0.041 | -0.1776 | No |
| 37 | E2F8 | E2F transcription factor 8 [Source:HGNC Symbol;Acc:HGNC:24727] | 5527 | 0.031 | -0.1874 | No |
| 38 | GJB5 | gap junction protein beta 5 [Source:HGNC Symbol;Acc:HGNC:4287] | 5542 | 0.029 | -0.1878 | No |
| 39 | GRB2 | growth factor receptor bound protein 2 [Source:HGNC Symbol;Acc:HGNC:4566] | 5697 | 0.016 | -0.1995 | No |
| 40 | TMED2 | transmembrane p24 trafficking protein 2 [Source:HGNC Symbol;Acc:HGNC:16996] | 5709 | 0.015 | -0.2000 | No |
| 41 | ETNK2 | ethanolamine kinase 2 [Source:HGNC Symbol;Acc:HGNC:25575] | 5897 | 0.002 | -0.2146 | No |
| 42 | PPARG | peroxisome proliferator activated receptor gamma [Source:HGNC Symbol;Acc:HGNC:9236] | 6006 | -0.005 | -0.2229 | No |
| 43 | PLK4 | polo like kinase 4 [Source:HGNC Symbol;Acc:HGNC:11397] | 6041 | -0.008 | -0.2254 | No |
| 44 | WNT7B | Wnt family member 7B [Source:HGNC Symbol;Acc:HGNC:12787] | 6230 | -0.023 | -0.2396 | No |
| 45 | PKD2 | "polycystin 2, transient receptor potential cation channel [Source:HGNC Symbol;Acc:HGNC:9009]" | 6254 | -0.024 | -0.2408 | No |
| 46 | GGNBP2 | gametogenetin binding protein 2 [Source:HGNC Symbol;Acc:HGNC:19357] | 6301 | -0.027 | -0.2438 | No |
| 47 | CCDC134 | coiled-coil domain containing 134 [Source:HGNC Symbol;Acc:HGNC:26185] | 6436 | -0.038 | -0.2534 | No |
| 48 | LHX3 | LIM homeobox 3 [Source:HGNC Symbol;Acc:HGNC:6595] | 6495 | -0.042 | -0.2569 | No |
| 49 | TTPA | alpha tocopherol transfer protein [Source:HGNC Symbol;Acc:HGNC:12404] | 6796 | -0.063 | -0.2789 | No |
| 50 | MAPK1 | mitogen-activated protein kinase 1 [Source:HGNC Symbol;Acc:HGNC:6871] | 6853 | -0.068 | -0.2817 | No |
| 51 | SETD2 | "SET domain containing 2, histone lysine methyltransferase [Source:HGNC Symbol;Acc:HGNC:18420]" | 6926 | -0.074 | -0.2856 | No |
| 52 | SP3 | Sp3 transcription factor [Source:HGNC Symbol;Acc:HGNC:11208] | 7033 | -0.082 | -0.2920 | No |
| 53 | CITED2 | Cbp/p300 interacting transactivator with Glu/Asp rich carboxy-terminal domain 2 [Source:HGNC Symbol;Acc:HGNC:1987] | 7100 | -0.087 | -0.2951 | No |
| 54 | CUL7 | cullin 7 [Source:HGNC Symbol;Acc:HGNC:21024] | 7105 | -0.088 | -0.2933 | No |
| 55 | PTK2 | protein tyrosine kinase 2 [Source:HGNC Symbol;Acc:HGNC:9611] | 7315 | -0.103 | -0.3072 | No |
| 56 | ETV2 | ETS variant transcription factor 2 [Source:HGNC Symbol;Acc:HGNC:3491] | 7365 | -0.108 | -0.3085 | No |
| 57 | FGFR2 | fibroblast growth factor receptor 2 [Source:HGNC Symbol;Acc:HGNC:3689] | 7374 | -0.108 | -0.3066 | No |
| 58 | LEF1 | lymphoid enhancer binding factor 1 [Source:HGNC Symbol;Acc:HGNC:6551] | 7467 | -0.116 | -0.3110 | No |
| 59 | CDX4 | caudal type homeobox 4 [Source:HGNC Symbol;Acc:HGNC:1808] | 7519 | -0.120 | -0.3122 | No |
| 60 | MEN1 | menin 1 [Source:HGNC Symbol;Acc:HGNC:7010] | 7542 | -0.122 | -0.3110 | No |
| 61 | NDP | norrin cystine knot growth factor NDP [Source:HGNC Symbol;Acc:HGNC:7678] | 7631 | -0.129 | -0.3149 | No |
| 62 | ADA | adenosine deaminase [Source:HGNC Symbol;Acc:HGNC:186] | 8105 | -0.163 | -0.3481 | No |
| 63 | HEY1 | hes related family bHLH transcription factor with YRPW motif 1 [Source:HGNC Symbol;Acc:HGNC:4880] | 8213 | -0.172 | -0.3524 | No |
| 64 | GHSR | growth hormone secretagogue receptor [Source:HGNC Symbol;Acc:HGNC:4267] | 8472 | -0.191 | -0.3681 | No |
| 65 | VASH1 | vasohibin 1 [Source:HGNC Symbol;Acc:HGNC:19964] | 8473 | -0.191 | -0.3635 | No |
| 66 | BMP5 | bone morphogenetic protein 5 [Source:HGNC Symbol;Acc:HGNC:1072] | 8488 | -0.193 | -0.3601 | No |
| 67 | MAP3K4 | mitogen-activated protein kinase kinase kinase 4 [Source:HGNC Symbol;Acc:HGNC:6856] | 8637 | -0.203 | -0.3669 | No |
| 68 | LEP | leptin [Source:HGNC Symbol;Acc:HGNC:6553] | 8740 | -0.212 | -0.3698 | No |
| 69 | PKD1 | "polycystin 1, transient receptor potential channel interacting [Source:HGNC Symbol;Acc:HGNC:9008]" | 8755 | -0.213 | -0.3659 | No |
| 70 | RBPJ | recombination signal binding protein for immunoglobulin kappa J region [Source:HGNC Symbol;Acc:HGNC:5724] | 8800 | -0.217 | -0.3642 | No |
| 71 | IL10 | interleukin 10 [Source:HGNC Symbol;Acc:HGNC:5962] | 8855 | -0.221 | -0.3632 | No |
| 72 | DEDD | death effector domain containing [Source:HGNC Symbol;Acc:HGNC:2755] | 8888 | -0.224 | -0.3605 | No |
| 73 | BSG | basigin (Ok blood group) [Source:HGNC Symbol;Acc:HGNC:1116] | 9043 | -0.235 | -0.3670 | No |
| 74 | PHLDA2 | pleckstrin homology like domain family A member 2 [Source:HGNC Symbol;Acc:HGNC:12385] | 9059 | -0.237 | -0.3625 | No |
| 75 | HAND1 | heart and neural crest derivatives expressed 1 [Source:HGNC Symbol;Acc:HGNC:4807] | 9082 | -0.238 | -0.3586 | No |
| 76 | NCOA1 | nuclear receptor coactivator 1 [Source:HGNC Symbol;Acc:HGNC:7668] | 9270 | -0.253 | -0.3673 | No |
| 77 | MME | membrane metalloendopeptidase [Source:HGNC Symbol;Acc:HGNC:7154] | 9287 | -0.255 | -0.3625 | No |
| 78 | ARNT | aryl hydrocarbon receptor nuclear translocator [Source:HGNC Symbol;Acc:HGNC:700] | 9427 | -0.267 | -0.3671 | No |
| 79 | ITGB8 | integrin subunit beta 8 [Source:HGNC Symbol;Acc:HGNC:6163] | 9442 | -0.268 | -0.3619 | No |
| 80 | BMP7 | bone morphogenetic protein 7 [Source:HGNC Symbol;Acc:HGNC:1074] | 9544 | -0.278 | -0.3632 | No |
| 81 | CYP27B1 | cytochrome P450 family 27 subfamily B member 1 [Source:HGNC Symbol;Acc:HGNC:2606] | 9582 | -0.282 | -0.3595 | No |
| 82 | HTRA1 | HtrA serine peptidase 1 [Source:HGNC Symbol;Acc:HGNC:9476] | 9692 | -0.291 | -0.3611 | No |
| 83 | MDFI | MyoD family inhibitor [Source:HGNC Symbol;Acc:HGNC:6967] | 9923 | -0.314 | -0.3717 | No |
| 84 | HSP90AB1 | heat shock protein 90 alpha family class B member 1 [Source:HGNC Symbol;Acc:HGNC:5258] | 10060 | -0.327 | -0.3747 | Yes |
| 85 | CDX2 | caudal type homeobox 2 [Source:HGNC Symbol;Acc:HGNC:1806] | 10078 | -0.328 | -0.3682 | Yes |
| 86 | NR2F2 | nuclear receptor subfamily 2 group F member 2 [Source:HGNC Symbol;Acc:HGNC:7976] | 10129 | -0.334 | -0.3643 | Yes |
| 87 | MAP2K1 | mitogen-activated protein kinase kinase 1 [Source:HGNC Symbol;Acc:HGNC:6840] | 10197 | -0.340 | -0.3615 | Yes |
| 88 | GCM1 | glial cells missing transcription factor 1 [Source:HGNC Symbol;Acc:HGNC:4197] | 10236 | -0.345 | -0.3563 | Yes |
| 89 | CTSB | cathepsin B [Source:HGNC Symbol;Acc:HGNC:2527] | 10313 | -0.352 | -0.3539 | Yes |
| 90 | PRDM1 | PR/SET domain 1 [Source:HGNC Symbol;Acc:HGNC:9346] | 10374 | -0.358 | -0.3502 | Yes |
| 91 | PLAC1 | placenta enriched 1 [Source:HGNC Symbol;Acc:HGNC:9044] | 10428 | -0.364 | -0.3457 | Yes |
| 92 | CASP8 | caspase 8 [Source:HGNC Symbol;Acc:HGNC:1509] | 10442 | -0.366 | -0.3381 | Yes |
| 93 | RBM15 | RNA binding motif protein 15 [Source:HGNC Symbol;Acc:HGNC:14959] | 10499 | -0.373 | -0.3337 | Yes |
| 94 | CCNF | cyclin F [Source:HGNC Symbol;Acc:HGNC:1591] | 10548 | -0.378 | -0.3285 | Yes |
| 95 | STK3 | serine/threonine kinase 3 [Source:HGNC Symbol;Acc:HGNC:11406] | 10656 | -0.388 | -0.3277 | Yes |
| 96 | DNAJB6 | DnaJ heat shock protein family (Hsp40) member B6 [Source:HGNC Symbol;Acc:HGNC:14888] | 10702 | -0.394 | -0.3219 | Yes |
| 97 | NOTCH2 | notch receptor 2 [Source:HGNC Symbol;Acc:HGNC:7882] | 10720 | -0.395 | -0.3139 | Yes |
| 98 | ADAM19 | ADAM metallopeptidase domain 19 [Source:HGNC Symbol;Acc:HGNC:197] | 10838 | -0.409 | -0.3134 | Yes |
| 99 | PLG | plasminogen [Source:HGNC Symbol;Acc:HGNC:9071] | 10939 | -0.423 | -0.3112 | Yes |
| 100 | GATA2 | GATA binding protein 2 [Source:HGNC Symbol;Acc:HGNC:4171] | 11058 | -0.439 | -0.3101 | Yes |
| 101 | STK4 | serine/threonine kinase 4 [Source:HGNC Symbol;Acc:HGNC:11408] | 11074 | -0.441 | -0.3009 | Yes |
| 102 | MED1 | mediator complex subunit 1 [Source:HGNC Symbol;Acc:HGNC:9234] | 11146 | -0.450 | -0.2958 | Yes |
| 103 | HSD17B2 | hydroxysteroid 17-beta dehydrogenase 2 [Source:HGNC Symbol;Acc:HGNC:5211] | 11148 | -0.451 | -0.2853 | Yes |
| 104 | BMPR2 | bone morphogenetic protein receptor type 2 [Source:HGNC Symbol;Acc:HGNC:1078] | 11174 | -0.454 | -0.2765 | Yes |
| 105 | FOSL1 | "FOS like 1, AP-1 transcription factor subunit [Source:HGNC Symbol;Acc:HGNC:13718]" | 11222 | -0.460 | -0.2693 | Yes |
| 106 | PPARD | peroxisome proliferator activated receptor delta [Source:HGNC Symbol;Acc:HGNC:9235] | 11301 | -0.471 | -0.2643 | Yes |
| 107 | ADM | adrenomedullin [Source:HGNC Symbol;Acc:HGNC:259] | 11543 | -0.506 | -0.2712 | Yes |
| 108 | RPS6 | ribosomal protein S6 [Source:HGNC Symbol;Acc:HGNC:10429] | 11662 | -0.526 | -0.2680 | Yes |
| 109 | NSDHL | NAD(P) dependent steroid dehydrogenase-like [Source:HGNC Symbol;Acc:HGNC:13398] | 11670 | -0.527 | -0.2561 | Yes |
| 110 | STC2 | stanniocalcin 2 [Source:HGNC Symbol;Acc:HGNC:11374] | 11746 | -0.540 | -0.2492 | Yes |
| 111 | EPAS1 | endothelial PAS domain protein 1 [Source:HGNC Symbol;Acc:HGNC:3374] | 11758 | -0.542 | -0.2373 | Yes |
| 112 | HIF1A | hypoxia inducible factor 1 subunit alpha [Source:HGNC Symbol;Acc:HGNC:4910] | 11833 | -0.556 | -0.2299 | Yes |
| 113 | BPTF | bromodomain PHD finger transcription factor [Source:HGNC Symbol;Acc:HGNC:3581] | 11870 | -0.565 | -0.2194 | Yes |
| 114 | HS6ST1 | heparan sulfate 6-O-sulfotransferase 1 [Source:HGNC Symbol;Acc:HGNC:5201] | 11932 | -0.579 | -0.2105 | Yes |
| 115 | LIF | LIF interleukin 6 family cytokine [Source:HGNC Symbol;Acc:HGNC:6596] | 12085 | -0.615 | -0.2079 | Yes |
| 116 | EGFR | epidermal growth factor receptor [Source:HGNC Symbol;Acc:HGNC:3236] | 12109 | -0.622 | -0.1950 | Yes |
| 117 | SNAI1 | snail family transcriptional repressor 1 [Source:HGNC Symbol;Acc:HGNC:11128] | 12365 | -0.696 | -0.1985 | Yes |
| 118 | PDGFB | platelet derived growth factor subunit B [Source:HGNC Symbol;Acc:HGNC:8800] | 12389 | -0.704 | -0.1836 | Yes |
| 119 | ASH1L | ASH1 like histone lysine methyltransferase [Source:HGNC Symbol;Acc:HGNC:19088] | 12405 | -0.708 | -0.1681 | Yes |
| 120 | SOCS3 | suppressor of cytokine signaling 3 [Source:HGNC Symbol;Acc:HGNC:19391] | 12453 | -0.725 | -0.1547 | Yes |
| 121 | JUNB | "JunB proto-oncogene, AP-1 transcription factor subunit [Source:HGNC Symbol;Acc:HGNC:6205]" | 12468 | -0.734 | -0.1384 | Yes |
| 122 | CITED1 | Cbp/p300 interacting transactivator with Glu/Asp rich carboxy-terminal domain 1 [Source:HGNC Symbol;Acc:HGNC:1986] | 12493 | -0.746 | -0.1227 | Yes |
| 123 | STC1 | stanniocalcin 1 [Source:HGNC Symbol;Acc:HGNC:11373] | 12622 | -0.809 | -0.1136 | Yes |
| 124 | PTGS2 | prostaglandin-endoperoxide synthase 2 [Source:HGNC Symbol;Acc:HGNC:9605] | 12641 | -0.820 | -0.0956 | Yes |
| 125 | HES1 | hes family bHLH transcription factor 1 [Source:HGNC Symbol;Acc:HGNC:5192] | 12676 | -0.849 | -0.0782 | Yes |
| 126 | DAZAP1 | DAZ associated protein 1 [Source:HGNC Symbol;Acc:HGNC:2683] | 12686 | -0.854 | -0.0588 | Yes |
| 127 | CCN1 | cellular communication network factor 1 [Source:HGNC Symbol;Acc:HGNC:2654] | 12802 | -0.970 | -0.0449 | Yes |
| 128 | CEBPB | CCAAT enhancer binding protein beta [Source:HGNC Symbol;Acc:HGNC:1834] | 12804 | -0.973 | -0.0219 | Yes |
| 129 | ZFP36L1 | ZFP36 ring finger protein like 1 [Source:HGNC Symbol;Acc:HGNC:1107] | 12888 | -1.244 | 0.0009 | Yes |
Table: GSEA details [plain text format]

  

Fig 2: GOBP\_PLACENTA\_DEVELOPMENT      
 Blue-Pink O' Gram in the Space of the Analyzed GeneSet

  

Fig 3: GOBP\_PLACENTA\_DEVELOPMENT: Random ES distribution      
 Gene set null distribution of ES for **GOBP\_PLACENTA\_DEVELOPMENT**
